# Patients’ Experiences of “Long COVID” in the Community and Recommendations for Improving Services: A Quality Improvement Survey

**DOI:** 10.1177/21501327211041846

**Published:** 2021-09-07

**Authors:** Mohammad Sharif Razai, Roaa Al-Bedaery, Laxmi Anand, Katherine Fitch, Hannah Okechukwu, Teniola M. Saraki, Pippa Oakeshott

**Affiliations:** 1 Population Health Research Institute, St George’s University of London, London, UK; 2 Wandsworth Primary Care Network, Chatfield Health Care, London, UK; 3 Imperial College London, London, UK

**Keywords:** community health, coronavirus, quality improvement, long covid, postacute COVID-19 syndrome

## Abstract

**Introduction:**

“Long COVID” is a multisystem disease that lasts for 4 or more weeks following initial symptoms of COVID-19. In the UK, at least 10% of patient report symptoms at 12 weeks following a positive COVID-19 test. The aims of this quality improvement survey were to explore patients’ acute and post-acute “long” COVID-19 symptoms, their experiences of community services and their recommendations for improving these services.

**Methods:**

Seventy patients diagnosed with COVID were randomly selected from 2 large socially and ethnically diverse primary care practices. Of those contactable by telephone, 85% (41/48) agreed to participate in the quality improvement survey. They were interviewed by telephone using a semi-structured questionnaire about community services for COVID-19 patients. Interviews lasted 10 to 15 minutes.

**Results:**

Forty-nine percent of patients reported at least 1 post-acute COVID-19 symptom. The most common were severe fatigue (45%), breathlessness (30%), neurocognitive difficulties (such as poor memory), poor concentration and “brain fog” (30%), headaches (20%), and joint pain (20%). Many patients felt isolated and fearful, with scant information about community resources and little safety netting advice. Patients also expected more from primary care with over half (56%) recommending regular phone calls and follow up from healthcare staff as the most important approach in their recovery.

**Conclusions:**

In line with patients’ requests for more support, the practices now routinely refer patients with long COVID to an on-site social prescriber who explores how they are getting on, refers them to the GP or practice nurse when required, and sign posts them to support services in the community.

## Introduction

“Long COVID” is a multisystem disease that lasts for 4 or more weeks following initial symptoms of COVID-19 (with or without a positive test).^1,2^ Long COVID describes signs and symptoms that continue or develop after acute COVID-19 (ie, 4 weeks after the initial illness). It includes both ongoing symptomatic COVID-19 (4-12 weeks) and post-COVID-19 syndrome (signs and symptoms that continue for more than 12 weeks).^3^ In the UK, 22% of patients report at least 1 symptom at 5 weeks following a positive COVID-19 test, and 10% at 12 weeks.^4^ The incidence of persistent symptoms for hospitalized patients and those attending specialist clinics is much higher ranging from 40 to 90%.^5^ Most studies have focused on hospital patients and little is known about the experiences of patients in the community.^6^ Our aim was to conducted a quality improvement survey to explore patients’ acute and post-acute “long” COVID symptoms, their experiences of community services and their recommendations for improving these services.

## Methods

In November 2020, 4 doctors and 2 medical students conducted telephone interviews with patients with suspected and/or laboratory-confirmed COVID-19 in 2 large, socially and ethnically diverse inner-city practices in London, each with around 12 000 registered patients. An initial electronic search revealed 130 and 150 patients in the 2 practices had been diagnosed with COVID-19. We randomly selected a total of 70 patients 4 weeks following their initial COVID-19 symptoms and telephoned them to ask whether they would agree to be interviewed about their experience of care after acute COVID-19, and recommendations for service improvements. Twenty-two patients were uncontactable, and 85% (41/48) of the remainder agreed.

Interviews were undertaken by telephone using a semi-structured questionnaire guide (see Appendix) designed to evaluate and improve services for long COVID patients. Interviews lasted about 10 to 15 minutes. The design of the interview questions and prompts were informed by current understanding of long COVID.^6,7^ Interviews were transcribed, subjected to thematic analysis and robustly discussed by the team to agree on interpretation of the data. Patients’ demographic details were extracted from their electronic health record. Data were collected as part of service improvement by the primary care sites. Informed consent was obtained from all participants.

## Results

**Figure 1.**
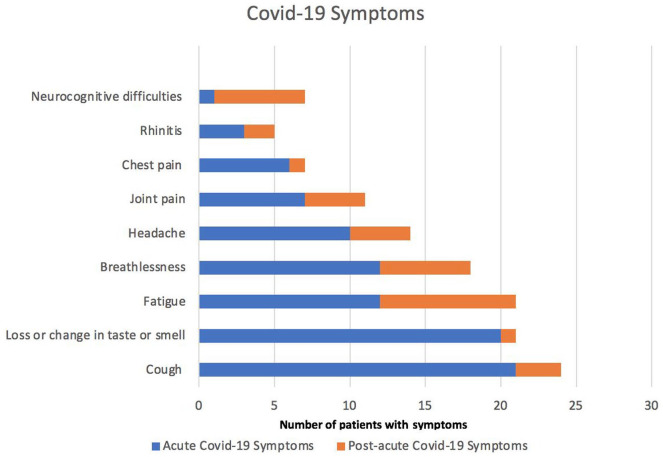
Reported COVID-19 symptoms during acute (blue) and post-acute phase (orange).

The demographic and clinical characteristics of the patients surveyed are summarized in Table 1. Forty-nine percent reported at least 1 post-acute COVID-19 symptom, of which the most common included severe fatigue (45%), breathlessness (30%), neurocognitive difficulties (such as poor memory), poor concentration and “brain fog” (30%), headaches (20%), and joint pain (20%; Figure 1).

**Table 1.** Demographic and Clinical Characteristics of the 41 Patients Interviewed.

Characteristics	Value
Age in years, mean (range)	49 (19-82)
Female	27 (66%)
Ethnicity	
Black	6 (15%)
White	23 (56%)
Asian	9 (22%)
Mixed	2 (5%)
Other	1 (2%)
Comorbidities	
Diabetes	8 (20%)
Hypertension	10 (24%)
Hyperlipidemia	5 (12%)
Hypothyroidism	3 (7%)
Obesity	11 (27%)
Asthma	7 (17%)
COPD	2 (5%)
Chronic kidney disease	1 (2%)
Depression	3 (7%)
Smoking	2 (5%)
Post-acute COVID-19 symptoms	
No symptoms	21 (51%)
1-2	13 (32%)
3 or more	7 (17%)
Quality of life worse	14 (34%)
Accessed GP Services	30 (73%)
Accessed other community services	7 (17%)

Four themes emerged out of the interviews: living with fear and uncertainty; the impact of long COVID on patients’ lives; experiences of accessing GP care; recommendations to improve services to support recovery from long COVID.

### Fear, Isolation, and Uncertainty Related to COVID-19

Several patients expressed their fear especially considering the uncertainty of COVID-19 and being left on their own without access to advice.

*“It’s a frightening experience, very isolating, I felt I was left on my own. It’d really help to get a call.” (35-year-old, female)*

*“No one checked how I was doing, no one called back, did not feel very supported. Very frightening time for us. Left on my own to cope and forgotten about. I thought I was going to die. I lost a family member to covid.” (55-year-old, female)*

Some patients mentioned multiple symptoms and their concern about which of them needed further investigation and examination.

*“I’ve lots of symptoms, and it was very good to talk to a doctor regularly to check which ones I should worry about or investigate.” (43-year-old, female)*

### Impact of Long COVID on Patients’ Lives

Many patients reported several symptoms that had had a severe impact on their lives both mental and physical.

*“Back to work but get easily tired, it’s the fatigue - unable to work full time now. My workplace is quite understanding, I work as community nurse. I live with my daughter.” (64-year-old, female)*

*“I don’t know where to start. . .Uncertainty is the word I would use about the whole thing. Enforcing social distancing in public transport is one thing, it stresses me out. Getting in touch with people more isolated than me during their illness is very important.” (39-year-old, female)*

### Experiences of Accessing GP Services for Long COVID

Some patients mentioned the difficulty of not being able to access face to face GP services during the pandemic and the delay to get an appointment.

*“It is a shame GP can’t see me face to face, it is very difficult to express everything over the phone.” (55-year-old, female)*

*“I prefer to see the doctor face to face but because of the situation it is ok. Having difficulty finding appointments. You have to call and wait a long time on the phone. I am very behind on this online stuff so I can only book by calling by phone.” (52-year-old, female)*

Other patients found telephone consultation helpful. However, these were mostly younger people in professional occupations.

*“Having the same doctor to talk to was very helpful. I found telephone consultations excellent.”* (35-year-old, male)

*“Very quick telephone consultation which was very important and helpful - it would be good to continue telephone consultation after covid*.” (31-year-old, male)

### Recommendations to Improve Primary Care Services to Support Patients’ Recovery From Long COVID

Patients recommended regular follow-up and phone calls from healthcare staff (56%) as the most important and helpful approach in their care.

*“It would be nice to get reassurance and speak to someone on a regular basis especially as my wife is diabetic so we know what we can do to help ourselves. Even if there is no remedy there must be something we could do. Just a bit more help from my doctors - that is all I ask for.” (56-year-old, male)*

Patients frequently mentioned reassurance and regular follow-up calls.

*“I would have liked a call from a doctor/nurse with advice and reassurance, especially on how family members can help without getting infected, as I was isolating from them in the same household.” (70-year-old male)*

*“If there was a GP who would have seen her to examine her and reassure me I would have felt so much more at ease.” (60 year old, male)*

Some patients reported a virtual support group as helpful. However, this was mostly the case for younger patients who had access to digital resources.

*“Having a virtual support group would be very helpful- a virtual patient group. A Facebook page for long covid can be helpful but it can also hinder, it can also send you off to different directions.” (25-year-old female)*

Better and more accessible information was also frequently mentioned as well as wider access to testing for COVID.

*“It was useful to be able to ring if I was worried. Once I coughed up blood, and it was good to talk to someone. It would have been useful to have more information about covid-19 from my GP.” (61, Female)*

*“Doctor should call us more and send information. We haven’t had much from anyone else. That is what we lack, even if it is just a phone call every now and again.” (56-year-old, male)*

*“Pre-emptively call patients. Making information more accessible to lay people. Positive experiences of how people are coping and recovering.” (25-year-old female)*

*“It was good to get the antibody tes. Make a test available for people.” (29-year-old female)*

*“Found the length of time to get a test annoying and no available antibody test which I would have liked.” (50-year-old, Male)*

## Discussion

Long COVID is a burgeoning global problem in the community.^6,8^ It is crucial to learn from our patients’ experiences in order to provide optimum care. Patients reported the difficulty of living with long COVID in an uncertain and constantly evolving situation. Our survey demonstrates that patients from a range of backgrounds expected more from primary care, suggesting regular follow-ups would be helpful in their recovery. In addition, many patients felt isolated and fearful, with scan information about community resources and little safety netting advice.

These findings have helped to change clinical practice and led to service improvements. In line with patients’ requests for more support, the practices now routinely refer patients with long COVID to an on-site social prescriber who explores how they are getting on, refers them to the GP or practice nurse when required, and sign posts them to support services in the community. In addition, patients who are unwell with acute COVID are able to have an urgent medical consultation and are routinely offered a pulse oximeter and ongoing support.

These findings of this quality improvement survey are supported in the literature with patients reporting a need for their GP at every stage of their illness,^9^ quoting a desire for someone to listen and acknowledge the impact of their symptoms. Other community-based studies have also reported fear and helplessness in long COVID patients.^6,9^ These feelings were compounded by uncertainty regarding the trajectory of their illness and markers of serious disease.^9^ Enabling self-management in the community is a key arm in the multidisciplinary approach to the management of long COVID.^10^ This can include education regarding self-monitoring, patient support groups, and direction regarding management of mental health. This could perhaps be undertaken by trained non-clinical staff such as social prescribers in the first instance, providing welfare calls with a clinician’s review where indicated. Management should also be based on supporting patient self-care and harnessing community resources.^11^

Limitations of this survey include the relatively small number of participants, but this is usual in many service evaluation projects. Findings may only apply to these London-based general practices and may not be widely applicable. However, findings may provide important teaching points for primary healthcare workers. Further studies are needed to improve primary care services for patients recovering from post-acute COVID-19 in the community.

## Appendix

### Semi-Structured Questionnaire

1. What physical and mental health symptoms of coronavirus did you have?
2. What physical and mental health symptoms of coronavirus do you still have?
3. What impact has coronavirus had on your life to date including your other medical conditions?
4. Tell me more about your home situation and how you are coping at the moment?
5. What was your experience of using GP and community services during acute illness and recovery from coronavirus?
6. How satisfied are you with GP and community services for coronavirus during your recovery?
7. Did you receive any coronavirus specific support or were you sign-posted to such services in the community during your recovery?
8. If you received specific community support during your recovery from coronavirus, what was your experience?
9. What did you find most useful and least useful during your illness and recovery from coronavirus?
10. What do you recommend needs to improve to make the experience of those recovering from COVID-19 better in the community?
